# MicroRNAs as Novel Biomarkers in Colorectal Cancer

**DOI:** 10.3389/fgene.2012.00180

**Published:** 2012-10-19

**Authors:** Sonja Hrašovec, Damjan Glavač

**Affiliations:** ^1^Department of Molecular Genetics, Institute of Pathology, Faculty of Medicine, University of LjubljanaLjubljana, Slovenia

**Keywords:** colorectal cancer, miRNAs, microsatellite instability, DNA methylation

## Abstract

MicroRNAs (miRNAs) play an important role in various physiologic and developmental processes and in the initiation and progression of cancer. This class of small, non-coding RNAs critically regulate gene expression at the post-transcriptional level and evidence suggests that they may function as both oncogenes and tumor suppressors. Colorectal cancer (CRC) is a major healthcare concern worldwide and in order to reduce CRC related deaths, research is aimed into the search for some novel screening approaches. In this sense, miRNAs are rapidly emerging as a novel class of biomarkers, with good potential as diagnostic and therapeutic targets. This review summarizes the recent findings of the clinicopathological relevance that miRNAs have in CRC initiation, development, and progress, highlighting their potential diagnostic, prognostic, and therapeutic use in CRC, focusing on the group of microsatellite instable and the group of hypermethylated CRCs, as well as discussing future prospects.

## Introduction

**Figure 1:**
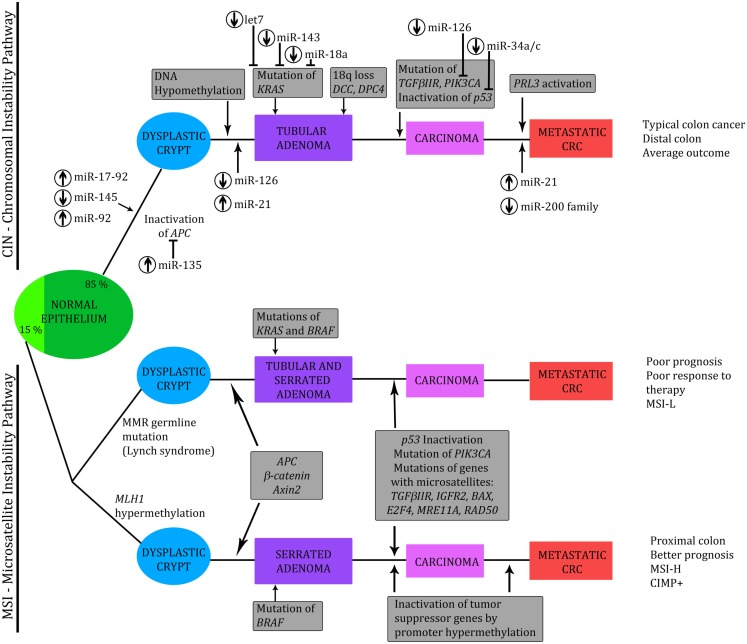
**Genetic model of the adenoma to carcinoma sequence**. Chromosomal instability (CIN) and microsatellite instability (MSI) pathways, with implicated third – CpG island methylator pathway (CIMP). Involved miRNAs are indicated, together with assigned miRNA UP-(↑) or DOWN-(↓) regulation. Modified from Slaby et al. (2009), Vilar et al. (2011), Valeri et al. (2009), and Zarate et al. (2012).

Cancer is the leading cause of death in economically developed countries and the second leading cause of death in developing countries. Colorectal cancer (CRC) is the third most commonly diagnosed cancer in males and the second in females, with over 1.2 million new cases each year. CRC incidence rates are rapidly increasing due to the effect of many risk factors, including smoking, physical inactivity, excess weight and obesity, red and processed meat consumption, and excessive alcohol consumption (Jemal et al., 2011). CRC is a major health problem. In order to find new diagnostic and therapeutic solutions that could help reduce CRC related deaths, it is important to understand the etiological and biological nature of CRC. Understanding the molecular genesis of CRC is a fundamental step in the identification of novel molecular targets that might be useful in defining the prognosis of CRC patients and tailoring their therapy (Valeri et al., 2009). Progression to CRC is considered a stepwise process, with an accumulation of various genetic and epigenetic alterations, leading to transformation from a normal cell to a premalignant tumor and finally to a malignant and potentially metastatic tumor (normal to adenoma to carcinoma sequence; Oberg et al., 2011). CRC develops through two main genetic pathways, characterized by different forms of genomic instability, as presented in Figure 1.

Most tumors (approximately 85%) are generated by the chromosomal instability (CIN) pathway and display marked cytogenetic abnormalities, aneuploidy, and allelic losses at multiple chromosomal arms. CIN is probably caused by various molecular mechanisms but the underlying genetic alterations are still poorly defined. This group is considered to be microsatellite stable (MSS). About 15% of colorectal carcinomas develop through the microsatellite instability (MSI) pathway. MSI results from defects in the DNA mismatch repair system (MMR). Molecularly different subgroups of CRC tumors: MSS and MSI cancers histologically appear similar but, clinically, they behave differently. MSI adenocarcinomas display distinctive pathologic features, such as proximal location, poor differentiation, frequent mucinous and medullary phenotype, and marked peritumoral and intratumoral lymphocytic infiltration. High microsatellite instable (MSI-H) cancers have a better prognosis: in general, they follow a more benign disease course; however, low MSI (MSI-L) cancers respond less well to chemotherapy (Lanza et al., 2007; Schepeler et al., 2008). MSI can occur through a germline mutation in MMR genes or, in most sporadic cases, when the promoter region of the genes in the MMR (usually *MLH1*) is silenced by hypermethylation of the CpG island. Analysis of methylation of CpG islands, as a mechanism of silencing genes in colon tumors, has resulted in the identification of the CpG island methylator phenotype, which seems to be complex and represents a third subclass of CRC tumors. This serrated pathway, also called CpG island methylator pathway (CIMP), usually occurs in the right colon, and seems to be governed by a progression of different molecular mechanisms to the classic adenoma carcinoma sequence (Cunningham et al., 1998, 2010; Toyota et al., 1999; Shen et al., 2007).

Human cancers thus comprise both genetic (inherited and acquired) and epigenetic alterations. Many tumor suppressor genes and oncogenes have been described and the discovery of new tumor markers continues at a rapid pace, also in CRC. A novel group of biomarkers, microRNAs (miRNAs), has recently been discovered. These molecules appear to be cell type and disease specific, unlike most other markers currently available. MiRNAs, as small regulatory non-coding RNA molecules, direct the binding of protein complexes to specific nucleic acid sequences and can therefore repress the expression of the target genes at the post-transcriptional level by interacting with the 3′ untranslated regions (UTRs) of specific mRNAs (Fazi and Nervi, 2008). miRNAs are believed to have an impact on laboratory medicine as new diagnostic and prognostic markers, as indicators of therapeutic response, and as targets of novel therapies (Bartels and Tsongalis, 2009).

In this review, we will focus on the critical role that miRNAs have in CRC, summarize recent findings concerning the clinicopathological relevance and potential diagnostic, prognostic, and therapeutic implications of miRNAs in CRC, briefly highlight their potential as biomarkers in the group of microsatellite instable (MSI) and group of hypermethylated CRCs, as well as discuss future prospects.

## miRNA Biogenesis

At the time of this review, 1921 mature human miRNA sequences were cataloged in the Sanger Institute miRBase (V18; http://www.mirbase.org/index.shtml, accessed on 29 June 2012) and over 6800 papers had been published in the PubMed database associating miRNA to cancer, including 339 publications concerning miRNA in CRC only.

MiRNAs are small, endogenous; single stranded RNA molecules that play important regulatory roles in animals and plants by targeting mRNAs for cleavage or translational repression of approximately aq third of all known protein-coding genes. They are transcribed as long primary transcripts – pri-miRNA, which undergo sequential processing mediated by two RNase III endonucleases, Drosha and Dicer. The process yields a ∼22-nucleotide long duplex miRNA: miRNA^*^. One strand of the duplex assembles into the RNA interference-effector complex RISC (RNA-induced silencing complex), whereas the other is generally degraded. The complex is then directed to partially complementary miRNA 3′UTRs by base pairing (Bartel, 2004; Mirnezami et al., 2009). By targeting mRNAs, they consequently inhibit mRNA translation and protein synthesis or cause mRNA degradation, depending on the imperfect pairing or exact match pairing to the mRNA, respectively. In other words – whether translational inhibition or mRNA degradation will occur depends on the degree of miRNA complementarity to the mRNA molecule. For a functional interaction between the miRNA and target sequence, only a limited number of complementary basepairs is required. The part of miRNA that exactly aligns to mRNA is called “the seed sequence” and is approximately 6–8 bases long (Bartels and Tsongalis, 2010). One miRNA can target about 200 mRNAs responsible for various proteins, while one mRNA can be controlled by several miRNAs (Lim et al., 2005). In order to characterize the function of miRNA, it is necessary properly to identify its target. The most commonly used approach is a bioinformatical search using various computational programs, such as TargetScan, miRanda, PicTar etc. (Lindow and Gorodkin, 2007; Kuhn et al., 2008; Saito et al., 2009).

## Altered miRNA Expression in Cancer

Each miRNA can regulate hundreds of different protein-coding genes. They have been shown to regulate several developmental and basic cellular processes, such as cell differentiation, growth, proliferation, and apoptosis. miRNA expression levels differ between healthy tissue and cancerous tissue; moreover inhibition of Drosha or Dicer leads to decreased miRNA expression and an enhancement of transformation and tumor growth (Williams, 2008). Over a decade ago, miRNAs were implicated in the initiation of chronic lymphocytic leukemia. The authors for the first time reported evidence of the involvement of miRNA genes in human tumors (Calin et al., 2002). Lu et al. (2005) subsequently analyzed the expression levels of more than 200 miRNAs across 334 primary tumors, healthy tissue, and cell lines of a wide range of different cancers and showed that the miRNA profile can better classify tumors from control healthy samples than the mRNA expression pattern. Since then, miRNAs have been shown to be involved in the cancerogenesis of a variety of cancers, including CRC, with a role as tumor suppressor genes or oncogenes (presented in Tables 1 and 2, respectively).

**Table 1:** **Tumor suppressor miRNAs differentially expressed in colorectal cancer compared to normal mucosa and their predicted targets**.

miRNA	Expression status in CRC (↑,↓)	Predicted target gene	Reference
**TUMOR SUPPRESSOR miRNAs**			
let-7 family	↓	*KRAS*	Akao et al. (2006a), Torrisani et al. (2007), Han et al. (2012), Wang et al. (2011a), Earle et al. (2010), Slattery et al. (2011)
miR-9	↓		Lujambio et al. (2008), Bandres et al. (2009)
miR-16	↓		Earle et al. (2010)
miR-26b	↓		Earle et al. (2010)
miR-34b/c	↓	*C-MYC, E2F2, CDK6*	Lujambio et al. (2008), Kalimutho et al. (2011), Toyota et al. (2008)
miR-129-2	↓		Bandres et al. (2009)
miR-137	↓	*CDC42*	Bandres et al. (2009)
miR-143	↓	*KRAS, DNMT3A, ERK5*	Michael et al. (2003), Akao et al. (2006b), Slaby et al. (2007), Wang et al. (2009), Motoyama et al. (2009), Chen et al. (2009), Earle et al. (2010), Ng et al. (2009)
miR-145	↓	*IRS-1, c-myc, SOX52*	Michael et al. (2003), Akao et al. (2006b), Slaby et al. (2007), Wang et al. (2009), Motoyama et al. (2009), Chen et al. (2009), Earle et al. (2010)
miR-148a	↓	*TGIF2*	Lujambio et al. (2008), Kalimutho et al. (2011)
miR-191	↓		Earle et al. (2010)
miR-192	↓		Earle et al. (2010), Balaguer et al. (2011)
miR-196a	↓		Earle et al. (2010)
mir-200c	↓	*ZEB1, ZEB2*	Chen et al. (2012a), Burk et al. (2008), Chen et al. (2012b)
miR-215	↓		Earle et al. (2010), Slattery et al. (2011)
miR-342	↓	*DNMT1*	Grady et al. (2008), Wang et al. (2011b)
miR-941	↓	*ADAM15*	Yan et al. (2011)
miR-1247	↓		Yan et al. (2011)

**Table 2:** **Oncogenic miRNAs differentially expressed in colorectal cancer compared to normal mucosa and their predicted targets**.

miRNA	Expression status in CRC (↑,↓)	Predicted target gene	Reference
miR-17-5p	↑		Lanza et al. (2007)
miR-20	↑		Lanza et al. (2007), Earle et al. (2010)
miR-21	↑	*PDCD4, MSH2, MSH6, PTEN, TPM1*	Slaby et al. (2007), Asangani et al. (2008), Schetter et al. (2008), Link et al. (2010), Valeri et al. (2010a), Slattery et al. (2011)
miR-25	↑		Lanza et al. (2007), Earle et al. (2010)
miR-31	↑		Earle et al. (2010)
miR-92-1	↑		Lanza et al. (2007)
miR-92-5	↑		Lanza et al. (2007)
miR-93-1	↑		Lanza et al. (2007)
miR-106a	↑		Lanza et al. (2007)
miR-135a	↑	*APC*	Earle et al. (2010)
miR-133b	↑		Earle et al. (2010)
miR-141	↑		Cheng et al. (2011)
miR-155	↑	*MLH1, MSH2, MSH6*	Valeri et al. (2010b)
miR-183	↑		Earle et al. (2010), Slattery et al. (2011)
miR-200b	↑		De Roock et al. (2010)
miR-203	↑		Earle et al. (2010)
miR-221	↑	*p53*	Pu et al. (2010)
miR-223	↑		Earle et al. (2010), Slattery et al. (2011)
miR-320	↑		Schepeler et al. (2008)
miR-498	↑		Schepeler et al. (2008)
miR-622	↑		Balaguer et al. (2011)
miR-1238	↑		Balaguer et al. (2011)

## Important Tumor Suppressor and Oncogenic miRNAs and Their Clinicopathological Relevance in CRC

Different studies have used a variety of techniques to examine the expression of selected miRNAs in order to determine their possible tumor suppressor or oncogenic function. Michael et al. (2003) were the first to show that miR-143 and miR-145 consistently display reduced steady-state levels of mature miRNA at the adenomatous and cancer stages of colorectal neoplasia, in comparison to healthy colon mucosa. Another study found that miRNA-143 and miRNA-145 expression levels were extremely reduced in colon cancer cells and by transfecting each precursor miRNA into the cells they achieved a significant growth inhibition in human colon cancer DLD-1 and SW480 cells. They also determined *ERK5* to be the target gene of miRNA-143 (Akao et al., 2006b). Several other researchers have confirmed those findings and correlated altered miR-143 and miR-145 expression with clinicopathologic features of CRC tumors. MiR-143 was found to be down-regulated in colon but not in rectal cancer and miR-145 expression was related to the cancer site. Researchers have shown that miR-143 functions as a tumor suppressor through inhibition of *KRAS* translation and that down-regulation of miR-143 drives tumor progression toward malignancy (Slaby et al., 2007; Chen et al., 2009; Motoyama et al., 2009; Wang et al., 2009). In the same study (Slaby et al., 2007), high expression of miR-21 was for the first time associated with lymph node positivity and the development of distant metastases in CRC. They thus managed significantly to correlate miR-21 up-regulation with CRC clinical stage (Slaby et al., 2007). Another group of researchers investigated the contribution of miR-21 in tumor cell invasion or intravasation. They confirmed for the first time an inverse correlation between miR-21 and Pdcd4 protein expression and proposed that miR-21 has a crucial role in post-transcriptional down-regulation of tumor suppressor *Pdcd4*, whose function is to stimulate cancer cell invasion, intravasation, and metastasis (Asangani et al., 2008). High miR-21 expression in tumors was associated with poor survival prognosis and poor therapeutic outcome in CRC, and in adenoma tissue it was found significantly enriched: 1.6-fold higher than in healthy colon mucosa (Schetter et al., 2008). Another group managed successfully to extract miRNA from stool and found miR-21 expression to be higher in stool samples from patients with colorectal neoplasia (adenocarcinomas and CRCs) than with subjects with normal colonoscopies. They showed that miRNAs could be easily, effectively, and reproducibly extracted from freshly collected stools and proposed that fecal miRNAs could serve as potential biomarkers in a non-invasive screening test for colorectal neoplasm (Link et al., 2010).

One of the first discovered miRNA families was the let-7 family and altered expression of these miRNAs has been described in many cancers (Torrisani et al., 2007). A study by Akao et al. showed reduced let-7 levels in CRC. When let-7 low-expressing human CRC cells were transfected with let-7a-1 precursor miRNA, this resulted in growth suppression and a decrease in RAS protein levels. At the same time, the levels of mRNA remained almost unchanged. Let-7 was suggested to act as a tumor suppressor in CRC (Akao et al., 2006a). Several other authors have confirmed the tumor growth suppression effect of let-7 family members, among them let-7c, which influences *MMP11* and *PBX3* gene expression. Let-7c was confirmed to have a tumor growth suppressor role but also found to be a tumor metastasis suppressor, which directly destabilizes the mRNA of *MMP11* and *PBX3* oncogenes (Han et al., 2012). Another group reported the synthetic let-7a capacity suppress expression of oncogene *NIRF* and thus cause a reduction in tumor growth. Their results open up the possibility of targeting *NIRF* in CRC therapy (Wang et al., 2011a).

Another important family of miRNAs is the miR-200 family. Members of this family, especially miR-200c, inhibit the metastatic ability of cancer cells by targeting the transcriptional repressor zinc-finger E-box binding homeobox 1 (*ZEB1*) in CRC. *ZEB1* is a crucial inducer of epithelial-mesenchimal transition, which promotes malignant tumor progression, invasion, and metastasis of tumor cells in various human cancers. Mir-200c was found to be down-regulated in CRC and when over-expression was regained, this significantly reduced the invasive potential of CRC cells (Burk et al., 2008; Chen et al., 2012a). Another group investigated the possible roles of miRNAs in regulating metastasis in paired CRC cells. Their results showed that over-expression of miR-200c was concurrent with down-regulation of *ZEB1* mRNA and protein. They demonstrated that miR-200c inhibits metastatic ability by targeting *ZEB1* in CRC cells and suggested that modulation of miR-200c with inhibitors or mimics could serve as a therapeutic tool for inhibiting metastasis in CRC. Their research provided a new insight into the development of miRNA-based cancer gene therapy for advanced CRC (Chen et al., 2012b).

## miRNA and Microsatellite Instability

As shown in Figure 1, CRC develops through two main genetic instability pathways, characterized by distinct pathologic features and clinical outcome. A high level of MSI is the molecular hallmark of a subset of CRCs and miRNAs have been shown to be useful in stratifying MSI-H CRCs from MSS CRCs. Lanza and colleagues evaluated miRNA expression in CRC samples and were the first to report the existence of differences in miRNA expression between MSS and MSI-H CRCs. Moreover, they suggested that the molecular classifier improves the separation between MSI and MSS cancer samples and that a combination of mRNA/miRNA expression signatures could provide improved stratification of tumor-associated character. Their study revealed that miR-17-5p, miR-20, miR-25, miR-92-1, miR-92-5, miR-93-1, and miR-106a were significantly up-regulated in MSS tumors compared to MSI-H. The above miRNAs are members of the mir-17-92 family, organized in three gene clusters. The chromosomes 13 gene cluster, which includes miR-17-92, had been previously found to be up-regulated in B-cell lymphoma. All this data suggests that members of miR-17-92 can act as oncogenes to promote cell growth and inhibit apoptosis, so up-regulation of these miRNAs may have a role in the more aggressive clinical behavior of MSS tumors (He et al., 2005; Lanza et al., 2007). Schepeler’s group investigated the expression profile of 315 human miRNAs in 10 normal mucosa samples and 49 stage II CRC samples differing with regard to microsatellite status and recurrence of disease. They showed that the microsatellite status of the majority of samples could be correctly predicted based on the miRNA expression profile. Furthermore, a biomarker based on miRNA expression profiles could predict recurrence of disease, indicating a potential role of miRNAs in determining tumor aggressiveness. The expression levels of miR-320 and miR-498, both included in the predictive biomarker, correlated with the probability of recurrence-free survival. In conclusion, they suggested that some miRNAs with prognostic potential could be of clinical importance (Schepeler et al., 2008).

Another study showed that over-expression of miR-155 significantly down-regulates the core MMR proteins, hMSH2, hMSH6, and hMLH1, inducing a mutator phenotype and MSI. They confirmed an inverse correlation between the expression of miR-155 and the expression of MLH1 or MSH2 proteins in CRC. In addition, a number of MSI tumors with unknown cause of MMR inactivation displayed miR-155 over-expression. Their results provide support for miR-155 modulation of MMR as a mechanism of cancer pathogenesis. This contributing role of miR-155 in the down-regulation of the core MMR proteins suggests that miR-155 expression might become an additional analytic test for exploring the etiology of MSI tumors when the standard tests do not provide a conclusive diagnosis (Valeri et al., 2010b). Another group of researchers managed to associate miRNA expression in CRC with MSI subgroups, including low MSI and hereditary non-polyposis CRC (HNPCC – Lynch syndrome)-associated cancers. They found 11 miRNAs (miR-183, -31, -20, -25, -92, -93, -17, -135a, -203, -133b, and -223) to be over-expressed in CRC relative to mucosa and nine (miR-192, -215, -26b, -143, -145, -191, -196a, -16, and let-7a) were under-expressed in CRC. The relative expression of miR-92, -223, -155, -196a, -31, and -26b were significantly different among MSI subgroups, and miR-31 and miR-223 were over-expressed in CRC of patients with HNPCC (Earle et al., 2010). Another group found eight miRNAs that appeared to be uniquely differentially expressed in MSI-H tumors compared to other tumor phenotypes, among others, miR-7, -183, -21, -215, -223 (Slattery et al., 2011). Balaguer et al. developed a miRNA-based predictor capable of differentiating between types of MSI in an independent sample set, in order successfully to discriminate between Lynch syndrome, sporadic MSI and sporadic MSS CRCs. They found a subset of nine miRNAs with deregulated expression, with miR-622, miR-1238, and miR-192 having the most differentially expressed expression between sporadic MSI tumors and Lynch syndrome tumors (Balaguer et al., 2011). MiR-21 over-expression in CRC has been reported several times, as discussed above. However, Valeri et al. were able to provide evidence that miR-21 directly targets the 3′UTR of MSH2 and MSH6 mRNA. MiRNA over-expression dramatically reduces the therapeutic efficacy of 5-FU via down-regulation of core mismatch repair recognition protein complex hMSH2-hMSH6, causing both primary and acquired resistance to 5-FU (Valeri et al., 2010a).

## Circulating Plasma miRNAs in CRC Patients

Tumor-associated miRNAs have been detected in the serum or plasma of patients suffering from breast and nasopharyngeal cancer, as well as CRC. Several studies have also reported that cell-free circulating miRNAs exist in serum and plasma. Chen and colleagues showed that serum miRNAs are stable and that their levels are reproducibly consistent among individuals of the same species. They also demonstrated that the serum miRNA expression profile can be used as a novel serum-based biomarker potentially offering more sensitive and specific tests than those currently available for early diagnosis of cancer (Chen et al., 2008). Researchers have confirmed miR-17-3p and miR-92 to be significantly elevated in patients with CRC. They have also shown the plasma levels of these markers to be significantly lower after surgical treatment. Using a miRNA detection approach in serum, they were able to discriminate CRC patients from control subjects with 89% sensitivity and 70% specificity (Ng et al., 2008). Another group of investigators proposed plasma miR-29a and miR-92a to be novel biomarkers for early detection of CRC. In their study, they were able to discriminate CRC patients from healthy controls by measuring plasma levels of miR-29a and miR-92a. Furthermore, they measured the expression of these miRNAs in plasma samples of 37 advanced adenomas, and found significantly elevated expression compared to those in normal controls. This study was one of the first to evaluate the diagnostic value of plasma miRNas in early detection of cancer (Huang et al., 2009). Pu and Huang et al. investigated the availability of direct amplification of miRNAs from plasma without RNA extraction. They revealed plasma levels of miR-221 to be a potential biomarker for differentiating CRC patients from controls. The elevated plasma miR-221 levels in their study on 103 CRC patients were shown to be a significant prognostic factor for poor overall survival and to be significantly correlated to p53 expression. Direct amplification of plasma miR-221 was therefore proposed as a potential non-invasive molecular marker for diagnosis and prognosis of CRC (Pu et al., 2010). Another group proposed miR-141 as a non-invasive biomarker in CRC. The authors identified and validated circulating miR-141 to be significantly associated with stage IV CRC in a large cohort of CRC plasma samples. They found miR-141 to improve the carcinoembryonic antigen role in detecting CRC patients with distant metastasis, as well associated high levels of miR-141 in the plasma of CRC patients with poor prognosis (Cheng et al., 2011). The most recent publication relating to plasma miRNAs reported the discovery of circulating miR-21 as a potential non-invasive marker in CRC. They found miR-21 in their study to be more up-regulated in stages III and IV than in stages I and II. Plasma miR-21 was able to differentiate CRC patients from controls with 90% specificity and sensitivity (Kanaan et al., 2012).

## miRNA and Hypermethylation Profile

DNA methylation is an important epigenetic mechanism that causes gene silencing. The so-called “CIMP pathway” has been shown to be implicated in a substantial proportion of CRC cases and has also been implicated in silencing of miRNA. Using the miRNA expression profile and DNMT1/DNMT3b double knockout cell lines, researchers were able to show that DNA hypermethylation contributes to the transcriptional down-regulation of miRNA in CRC. They found that the expression of about 10% of miRNAs was regulated by DNA methylation. In addition, neither 5-aza-2′deoxycytidine (5-Aza-CdR) treatment nor deletion of *DNMT1* alone restores the miRNA expression profile seen in the double knockout cell line, suggesting that miRNA expression was tightly controlled by DNA methylation and partial reduction was not efficient for miRNA re-expression (Han et al., 2007). Moreover, with drug treatment of lymph node derived metastatic cancer cells with a DNA demethylating agent, several miRNAs were reactivated. Among them, miR-148a, miR-34b/c, and miR-9 were found to undergo specific hypermethylation-associated silencing in cancer cells compared to normal tissue. The consequence was inhibited tumor cell motility, reduced tumor growth, and inhibited metastasis formation, with an associated down-regulation of miRNA oncogenic target genes, such as C-MYC, E2F2, CDK6, and TGIF2. Their findings indicate that DNA methylation-associated silencing of tumor suppressor miRNAs contributes to the development of human cancer metastasis (Lujambio et al., 2008). In addition, some other groups have investigated the hypermethylation of miR-34b/c in tumor DNA derived from feces and correlated it with clinicopathological features. They explored whether miR-34b/c could be used as a possible screening marker for the detection of malignant cancer cells in feces as a novel non-invasive method. They found that 75% (21/28) of samples had hypermethylated miR-34b/c. They proposed miR-34b/c to be an ideal candidate early screening marker in non-invasive fecal-DNA-based testing. In addition, they also succeeded in correlating the epigenetic silencing of miR-148a with a poor prognosis (Kalimutho et al., 2011). Another group investigated miR-34b and miR-34c as two components of the p53 network. Methylation of these two miRNAs was present in all cell lines investigated and in 90% (101/110) of primary CRC tumors inspected, but not in normal colonic mucosa. They found miRNA-34b and miR-34c to be epigenetically silenced in CRC. Their results also showed that transfection of precursor miR-34b and miR-34c into CRC cells induced dramatic changes in the gene expression profile. They found the miR-34b/c CpG island to be a bidirectional promoter driving expression of miR-34b/c genes and, due to the high frequency and tumor-specificity of miR-34b/c methylation, they proposed that it could serve as a useful tumor marker and important target in anticancer therapy (Toyota et al., 2008).

Grady and collaborators found that the expression of miR-342, a miRNA encoded in an intron of the EVL gene, was commonly suppressed in CRC. They found methylation at the EVL/miR-342 locus to be present in the majority of CRCs and even in more than half of adenomas, indicating that CpG island methylation of the upstream region of EVL is an early event in colorectal carcinogenesis. Furthermore, reconstitution of hsa-miR-342 in the CRC cell line HT-29 induced apoptosis, suggesting that this miRNA could function as a proapoptotic tumor suppressor. These results show that epigenetic alterations of host genes could be a mechanism of silencing intronic miRNA (Grady et al., 2008). One of the first miRNAs reported to be involved in CRC tumorigenesis was miR-143. A later study elucidated a tumor-suppressive role of miR-143 in the epigenetic aberration of CRC. The authors defined *DNMT3A* as a potential target of miR-143 and therefore proposed miR-143 as a regulator of *DNMT3A* expression in CRC. Restoration of miR-143 expression in colon cell lines decreased tumor cell growth and soft-agar colony formation, and down-regulated *DNMT3A* expression on both mRNA and protein levels (Ng et al., 2009). Bandres and colleagues used a sequential approach to identify tumor suppressor miRNAs that are silenced through aberrant epigenetic events in CRC. They first searched for down-regulated miRNAs that are located around/on a CpG island. They then treated cell lines with DNA methyltranspherase inhibitor in order to restore miRNA expression, and afterward checked for methylated promoter regions in the investigated genes. Using this approach, they identified down-regulation of miR-9-1, miR-129-2, and miR-137 and their expression was found to be in inverse correlation with their methylated promoter. Furthermore, methylation of miR-9-1 was associated with the presence of lymph node metastasis. Their experiment demonstrated that aberrant DNA methylation induces silencing of miRNA (Bandres et al., 2009). Another group investigated the expression of miR-342 in CRC in correlation with over-expressed *DNMT1*. They showed that miR-342 was down-regulated in CRC tissues and cell lines and that restoration of miR-342 resulted in a dramatic reduction of the expression of *DNMT1* at both messenger RNA and protein levels, by directly targeting its 3′UTR. This in turn reactivated *ADAM23, Hint1, RASSF1A*, and *RECK* genes via promoter demethylation. They proposed a new mechanism for the regulation of *DNMT1* and aberrant DNA hypermethylation in CRC, as well as demonstrating that miR-342 may act as a tumor suppressor gene in CRC development (Wang et al., 2011b). A recent study systematically searched for candidate miRNAs regulated by DNA methylation in CRC cell lines. They established a comprehensive catalog consisting of 64 epigenetically regulated miRNAs, of which 18 were shown to have an expression level consistent with their methylation status. Finally, the same authors performed a functional study to discover whether miR-941 and miR-1247 have an impact on cell growth and migration in CRC cell lines. They provided a reliable, systematic strategy for identifying new epigenetically regulated miRNAs involved in CRC progression (Yan et al., 2011).

## miRNA and *KRAS*

miRNA post-transcriptional inhibition depends on sequence complementation to 3′UTR of mRNA. Mutations in this region could interfere with miRNA inhibition and if these mutations occur in oncogenes, they may affect the important mechanism of inhibition in tumor cells. *KRAS* is a member of the Ras family of proteins, which regulates signaling pathways involved in cellular proliferation, differentiation, and survival. Bandres et al. searched for miRNA de-regulation patterns in patients carrying *KRAS* mutations and found that CRC cell lines with *KRAS* mutations showed over-expression of miR-9, miR-95, miR-148a, miR-190, and miR-372 in relation to the human normal colon cell line. The same study reported lower over-expression of the same miRNAs in CRC cell lines with mutations in *BRAF*. Activating mutations in *BRAF* are also considered able to activate the RAS/RAF/MEK/ERK pathway, consequently increasing cell proliferation but suppressing the inhibition of apoptosis. The authors summarize that altered miRNA expression of some miRNAs may deregulate cancer-related genes, such as *KRAS* and *BRAF* (Bandrés et al., 2006). Chen and colleagues noted an inverse correlation in KRAS protein levels in relation to miR-143 expression and a possible role of miRNAs in a tumor suppressor effect. They succeeded in experimentally validating *KRAS* as an miR-143 target, since in *in vitro* experiments, miR-143 directly bound to 3′UTR of the *KRAS* transcript (Chen et al., 2009).

In the most recent paper, the authors provided evidence that an increased level of miR-200b in *KRAS* mutated tumors is associated with good progression free survival. Patients with an activating mutation in *KRAS* were considered to be resistant to anti-EGFR therapy. They suggested that a codon 13 *KRAS* mutation has distinct clinical behavior and is not associated with anti-EGFR resistance (De Roock et al., 2010). Another recently published study proposed that elevated expression of miR-200b may be useful for identifying patients with mutated *KRAS* who would benefit from anti-EGFR therapy (Mekenkamp et al., 2012).

## Conclusion and Future Prospects

Since the first miRNA was identified in 1993, researchers have aimed for better understanding of the miRNA function in mammals. MiRNAs are easier and more appropriate to handle than mRNA, which gives them potential value in patient management. They clearly have a major impact on the initiation and CRC progression but in order to use them as diagnostic, prognostic, or even therapeutic targets, it is essential to establish and validate reliable, accurate, and standardized assays for the detection of aberrant miRNA expression. Secondly, when a therapeutic approach considered, overcoming the problem of administering the therapeutic to a target cell, avoiding contamination of other cells and organs, in order to minimize side effects, will be quite challenging. Although there are many obstacles to overcome, miRNAs have great future prospects as tumor markers in cancer classification, early detection, prognosis prediction, and therapeutic decision-making.
